# Acute Rejection in Renal Transplant Patients of a Hospital in Bogota, Colombia

**Published:** 2016-08-01

**Authors:** P. García, M Huerfano, M Rodríguez, A Caicedo, F Berrío, C Gonzalez

**Affiliations:** 1 *San Ignacio University Hospital*; 2 *Pontificia Universidad Javeriana*

**Keywords:** Kidney transplantation, Renal insufficiency, chronic, Kidney failure, chronic, Graft rejection, Graft survival, Incidence

## Abstract

**Background:**

Renal transplantation is the best treatment for end stage renal disease. Acute graft rejection is one of the main complications and may influence graft survival.

**Objective:**

To determine the incidence and features of acute cellular rejection (ACR) episodes confirmed by biopsy.

**Methods:**

We studied a cohort of 175 patients who underwent renal transplantation between 2004 and 2012 to determine the cumulative incidence of ACR confirmed by biopsy and to identify the associated risk factors using multivariate analysis.

**Results:**

The one-year patient survival was 96.6%; the graft survival was 93.7%. The incidence of ACR within one year was 14.3%, of which 46% were observed within 6 months following transplantation. The most frequently observed ACR type was 1B according to the Banff classification system (42%). A relationship between ACR and receipt of a kidney from expanded criteria donors was observed, both in univariate and adjusted multiple log-binomial regression analyses, but only 6.3% of patients received extended criteria donor kidneys. No other relationships between variables were found.

**Conclusion:**

ACR frequency in this study was similar to that of other cohorts reported previously. We need a bigger sample of renal transplants from expanded criteria donors, PRA and DSA test to support the results.

## INTRODUCTION

Acute graft rejection is a main complication of renal transplantation. The last 20 years have seen a significant decrease in acute cellular rejection (ACR), thanks to the introduction of more effective immunosuppressant therapies [1]. However, the reduction in ACR incidence has not been associated with increased long-term survival of renal transplants [2], and acute and chronic antibody-mediated rejection still plays a major role in renal graft loss [3].

Identified risk factors for ACR development include low histocompatibility between donor and recipient, the age of donor and recipient, ethnicity, sex, ischemia time, delayed graft function, graft non-adherence, and reduced immunosuppression, among other things [4].

The Organ Procurement and Transplantation Network and the United Network for Organ Sharing (OPTN/UNOS) state that ACR affects between 12% and 18% of transplant recipients who receive live donor kidneys and 14% to 30% of transplant recipients who receive deceased donor kidneys, particularly within six months from transplantation. Currently, ACR is responsible for 11% to 16% of graft loss within a year from the procedure, and 7% to 11% after one year [5]. T-cells reacting against expressed histocompatibility antigens are detected in the kidney tubules and interstitium in 45% to 70% of all cases, in vessels in 30% to 50% of all cases, and in the glomerulus in 2% to 4% of all cases, depending on the center where biopsies are performed [6].

Gamarra and collaborators described clinical features of rejection in 83 renal transplant recipients between 1981 and 1990 (102 months in total) at the Ramón González de Valencia University Hospital, Bucaramanga, Colombia. Observations for this group included 66 rejection episodes, of which 1 (1.5%) was hyperacute, 2 (3.0%) were accelerated, 53 (80.4%) acute, and 10 (15.1%) were chronic. ACR developed within 30 days from transplantation in 54.7% of cases. At the time these observations were made, our current histological classifications and immunosuppression techniques were not available. The study showed that, in patients with kidneys from deceased donors, the incidence of rejection decreased from 74% to 50% after administration of cyclosporine [7]. Recently, another study in Colombia conducted by Cubillos and collaborators found an 18.3% incidence of ACR one year after transplantation in a group of 160 patients [8]. We conducted the current study to establish the incidence, clinical features, and risk factors for ACR in 175 renal transplant recipients.

## PATIENTS AND METHODS

After authorization by the institutional ethics committee (approval number 2013/32), a retrospective cohort study was carried out at the Renal Transplantation Unit of the San Ignacio University Hospital (HUSI) in Bogotá, Colombia. The main objective was to determine the incidence and risk factors for ACR confirmed by biopsy. We excluded antibody-mediated rejection from the analysis. The study included 175 patients who underwent renal transplantation from either deceased or living donors and had at least a year of post-operative follow-up by March 2013. The diagnosis of acute rejection was confirmed by histology of graft biopsies, according to the Banff 2007 classification, whenever creatinine levels surpassed the basal level by 25% [9]. All graft biopsies per protocol were processed with four standard stains (periodic acid-shift, methenamine silver, trichrome, and hematoxylin-eosin), direct immunofluorescent (IgG, IgM, IgA, albumin, fibrinogen, and complement C3, C4, and C4d), and electron microscopy. In cases of suspected infection by polyomavirus, SV40 immunohistochemistry was processed.

Statistical Analysis

A database was created using Microsoft^®^ Excel 2011. Statistical analyses were performed using STATA^®^ ver 12.0. Continuous variables were expressed as mean or median with standard deviation, range, or interquartile range, as required. A bivariate analysis was performed using a χ^2^ or Fisher’s exact test for dichotomous variables. A *Student’s t* test was performed for normally distributed quantitative variables. Non-parametric tests were performed for variables with non-normal distributions. A multivariate analysis was performed using variables that were clinically and statistically associated with rejection outcomes, in order to identify risk factors through multiple log-binomial regression analysis to obtain relative risk.

## RESULTS

Between June 2004 and March 2012, 175 renal transplants were performed at the HUSI, Bogotá, Colombia. Most recipients were males (66.3%) with a mean±SD age at transplantation of 46.1±13.1 years; 1.7% of patients were Afro-Colombian. Of the patients in this cohort, 8.6% received a second transplant. Among the most frequent causes of chronic kidney disease (CKD) were hypertension (28.6%), diabetes mellitus (DM) (10.9%), and glomerulonephritis (18.3%). In 20% of patients, the cause of CKD was unknown. The median time of dialysis was 4.75 (range: 0.1–20) years; the predominant pre-transplant support therapy was hemodialysis.

The most frequently observed HLA phenotypes were A2 and A24, B35 and B7, and DR4 and DR1. In 85% of patients, no record of a panel reactive antibody (PRA) test was found. This paraclinical test was not available at the institution when the first patients in this study were treated. Of the 27 patients with PRA tests, 11 had a value >20%. The mean cold ischemia time was 12.2 hours. The most frequently used induction therapy drug was basiliximab (Table 1).

**Table 1:** Recipient data

Parameter	n (%)
Number of patients	175 (100)
Male	116 (66.3)
Blood type	
O	104 (59.4)
A	54 (30.9)
B	15 (8.5)
AB	2 (1.1)
CKD cause	
Hypertension	50 (28.6)
GMN	32 (18.3)
DM2	19 (10.9)
First transplant	160 (91.4)
CMV receptor IgG	163 (93.1)
Induction therapy	
Basiliximab	122 (69.7)
Thymoglobulin	37 (21.1)
Daclizumab	6 (3.4)
None	10 (5.7)

CKD: Chronic kidney disease; GMN: Glomerulonephritis; DM2: Diabetes mellitus type 2; CMV: Cytomegalovirus

A total of 168 transplants involving deceased donors and seven involving living donors were performed. Deceased donors were mostly male, with a median (IQR) age of 39 (24–48) years. The main cause of death was traumatic brain injury. A total of 11 (6.3%) cases involved expanded criteria donors. Expanded criteria donors were defined as age >60 years, or age 50–59 years plus two of the following: cerebrovascular accident as the cause of death, preexisting hypertension, or serum creatinine >1.5 mg/dL. The median for histocompatibility mismatch was 3; 5.7% had a mismatch of 6.

Results after One Year

The one-year patient survival rate was 96.6%; the one-year graft survival rate was 93.7%. Six patients died within the first year after transplantation. The causes of death were one case each of pulmonary thromboembolism, acute myocardial infarction, alveolar hemorrhage, abdominal aortic aneurysm, retroperitoneal hematoma, and hemorrhagic post-operative complications. The median (IQR) modification of diet in renal disease equation 4 (MDRD4)-calculated glomerular filtration rate after one year was 61.11 (27.5) mL/min/1.73 m^2^. The most common immunosuppressive regimens were cyclosporine, mycophenolate, and prednisone, followed by tacrolimus, mycophenolate, and prednisone. The median amount of proteinuria one year post-transplantation was 265 (range 22–3770) mg in 24 hours. Delayed graft function was observed in 14% of patients. The median (IQR) glomerular filtration rate calculated using the Cockroft-Gault equation was 64.71 (17.93) mL/min/1.73 m^2^. The same parameter calculated using MDRD4 gave a median (IQR) of 62.74 (25.02) mL/min/1.73 m^2^.

During the 4.8-year (range: 1.06–7.86 years) follow-up period, 78 rejection episodes were observed in 57 patients; two episodes were observed in 15 patients and four had >2 episodes. The incidence of ACR corroborated by biopsy after one year was 14.3%. The median (IQR) time for the first rejection episode was 110 (117) days. The most frequent ACR type, following the Banff 2009 classification, was 1B (42%), followed by 1A (27.5%). Most patients responded favorably to corticosteroid therapy.

Immunosuppressant drug concentrations were assessed when the first rejection episode occurred, revealing that 62% of patients were taking low drug concentrations during the episode.

The bivariate analysis revealed a correlation between ACR and receiving kidney from an expanded criteria donor (RR: 3.72, 95% CI: 1.73–8.02). Other factors such as recipient age (p=0.44), cold ischemia time (p=0.29), delayed graft function (p=0.12), degree of histocompatibility mismatch (p=0.43), donor type (deceased or living donor) (p=0.26), positive PRA (p=0.13), and thymoglobulin-mediated induction (p=0.06) were not statistically linked to ACR.

Multivariate analysis with multiple log-binomial regression was performed taking into account the recipient age, donor age, cold ischemia time, type of donor (deceased or living), expanded criteria donor, delayed graft function, thymoglobulin induction, and histocompatibility mismatch. Interaction between recipient age and expanded criteria donor, delayed graft function, and thymoglobulin use was also analyzed with regard to ACR development. Receiving a kidney from an expanded criteria donor correlated with ACR development (adjusted RR: 3.75, 95% CI: 1.80–7.81), however, only 11 of 175 patients received extended criteria donor kidneys (6.3%), and some important risk factors could not be studied, because only 27 of 175 patients had a PRA test. 

## DISCUSSION

HLA class I and II haplotypes of both donors and recipients in this study were similar to those reported in a recent publication by Arrunátegui, *et al*, which was also carried out in Colombia [10]. Within our Colombian cohort, the cause of ACR was not clearly identified in 66% of transplant patients. Similar findings were made in other Latin American countries [11]. The percentage of patients with glomerulonephritis or DM type 2 as the cause of CKD was similar to information published in the UNOS records [5].

The median dialysis time prior to transplantation in our cohort was 4.75 years, similar to the OPTN/UNOS records, which showed that 38% of recipients of kidneys from deceased donors underwent dialysis for at least five years. A total of 6% of patients underwent pre-emptive transplantation, a figure comparable to the 7% published in OPTN/UNOS records. Most patients (74.8%) received anti-IL2R induction therapy, according to their low-risk status. With a mean age of 36 years, deceased donors included in our study were slightly younger than those in other countries. The majority were male with traumatic brain injury as the main cause of death [5]. Likewise, recipients in our study were comparatively young, with a mean age of 46 years.

The cumulative incidence of ACR observed in our study was 14.3%, comparable to results in related literature [1, 5, 8]. The only variable found to correlate with ACR was receipt of a kidney from an expanded criteria donor (adjusted RR: 3.75). No other relationships between variables were observed, perhaps due to the limited sample size. 

We observed that 14.3% of patients had delayed graft function, a figure much lower than the 23% published in the 2012 OPTN/UNOS records. This difference may be due to the fact that our study involved younger donors, most of whom were not expanded criteria donors (93.7%) and due to shorter ischemia time (12.2 hours on average) compared with the OPTN/UNOS registry. ACR incidence after one year was similar to that in OPTN/UNOS—46% of rejection episodes occurred during the first six months. Similarly, a recently published study by Wu and colleagues reported that most ACR episodes (79.6%) occurred within six months following transplantation [12]. However, a remarkable 40% of rejection episodes in our study occurred more than one year from transplantation, which may be related to non-adherence to treatment regimens. Indeed, 62% of patients in our study were taking low concentrations of immunosuppressant drugs at the time of rejection, which could be due to non-adherence or to a prescribed lowered dosage. 

The study by Sellares, *et al*, showed that non-adherence to drug treatment was ten times greater among patients with graft failure compared with those without graft failure (32% *vs*. 3%). That study also revealed that the loss of renal grafts was due to humoral/mixed rejection with 47% of patients who lost grafts not adhering to treatment [3]. A study conducted by Gupta, *et al*, suggests that one feature of late antibody-mediated rejection is decreased immunosuppressant therapy prescribed by clinicians, as observed in 69% of patients [13]. In our study, 70% of ACR episodes were classified as 1A or 1B, 16% were borderline, and the rest fitted into Banff categories 2 and 3. Our cohort had a humoral rejection incidence of 0.3% after one year.

Several factors increasing ACR risk have been described, including younger recipients, low immunosuppressant levels, delayed graft function, prior sensitization to major histocompatibility complex antigens, and non-adherence to immunosuppressant therapy [4, 14-16]. In contrast, delayed graft function and cold ischemia time were not associated with greater ACR risk in our cohort, similar to other studies [3, 4].

Several recent studies highlight the links between ACR and donor specific antibody (DSA) production and with graft survival [17, 18]. Wu, *et al*, reported that, regardless of the ACR type, graft survival is invariably affected. Type I and II/III ACR patients were contrasted with a control group, showing reduced graft survival in patients that developed ACR of any type after an eight-year follow-up. Also, there was a significant decrease in graft survival among ACR patients who developed the condition after six months of transplantation, compared to those who developed ACR early (63.6% and 87.4%, respectively; p<0.001) [12]. Similarly, Dorje, *et al*, reported decreased graft survival in patients with late-developing antibody-mediated ACR, compared to early-developing cases (40% and 75%, respectively). Factors most closely related to the development of antibody-mediated ACR were a greater *de novo* DSA production incidence, non-adherence to therapy, suboptimal immunosuppressant therapy, and younger age [19].

DeVos, *et al*, showed that 24% of 503 transplant patients included in the study developed *de novo* DSA. Those with DSA were more likely to have ACR than those without DSA (35% *vs*. 10%; p<0.001) and more likely to experience antibody-mediated rejection (16% *vs*. 0.3%; p<0.001). After a 31-month follow-up period, survival of the graft was lower in DSA-positive patients [20]. The findings described above suggested that patients with ACR or antibody-mediated ACR had a lower graft survival. This was exacerbated when ACR developed late. Moreover, decreases in graft survival were closely linked to non-adherence to therapy, suboptimal immunosuppressant therapy, and *de novo* DSA production. Thus, we emphasize the importance of strict adherence to drug therapy for transplant patients, as this may affect graft survival.

The present study identified factors associated with ACR. Data on possible outcomes such as ACR and delayed graft function as well as patient and graft survival were similar to previous international reports. Variables associated with acute rejection in the sampled population were not different from those observed in other countries. 

Patients receiving renal transplants from expanded criteria donors had a high risk of ACR. We need a larger sample of renal transplants from expanded criteria donors, PRA and DSA test to further support our results. Unfortunately, we did not have data on immunosuppressant concentrations in patients who did not develop ACR in order to contrast with patients that did develop the condition.

About 62% of patients in our study were taking low concentrations of immunosuppressant drugs at the time of rejection, which could be due to non-adherence or to a prescribed lowered dosage. Patients should rigorously adhere to an established course of therapy and keep immunosuppressant drug concentrations stable, especially one year post transplantation, since 40% of ACR episodes occurred beyond one year from transplantation, a situation that may negatively influence graft survival. 
